# Influenza, Updated COVID-19, and Respiratory Syncytial Virus Vaccination Coverage Among Adults — United States, Fall 2023

**DOI:** 10.15585/mmwr.mm7251a4

**Published:** 2023-12-22

**Authors:** Carla L. Black, Jennifer L. Kriss, Hilda Razzaghi, Suchita A. Patel, Tammy A. Santibanez, Mehreen Meghani, Ashley Tippins, Shannon Stokley, Kevin Chatham-Stephens, Nicole F. Dowling, Georgina Peacock, James A. Singleton

**Affiliations:** ^1^Immunization Services Division, National Center for Immunization and Respiratory Diseases, CDC; ^2^Division of Readiness and Response Science, Office of Readiness and Response, CDC.

**Summary**

**What is already known about this topic?**

The Advisory Committee on Immunization Practices recommends that all adults receive influenza and COVID-19 vaccines, and those aged ≥60 years may receive respiratory syncytial virus (RSV) vaccine during the 2023–24 respiratory virus season.

**What is added by this report?**

By December 9, 2023, an estimated 42.2% and 18.3% of adults aged ≥18 years had received influenza and updated 2023–2024 COVID-19 vaccine, respectively; 17.0% of adults aged ≥60 years had received RSV vaccine. Many adults who had not received the vaccines reported being open to vaccination.

**What are the implications for public health practice?**

Strong provider recommendations for and offers of vaccination could increase influenza, COVID-19, and RSV vaccination coverage. Immunization programs and vaccination partners might benefit from using these within-season data to understand vaccination patterns in their jurisdictions to strengthen vaccination activities.

## Abstract

During the 2023–24 respiratory virus season, the Advisory Committee on Immunization Practices recommends influenza and COVID-19 vaccines for all persons aged ≥6 months, and respiratory syncytial virus (RSV) vaccine is recommended for persons aged ≥60 years (using shared clinical decision-making), and for pregnant persons. Data from the National Immunization Survey-Adult COVID Module, a random-digit–dialed cellular telephone survey of U.S. adults aged ≥18 years, are used to monitor influenza, COVID-19, and RSV vaccination coverage. By December 9, 2023, an estimated 42.2% and 18.3% of adults aged ≥18 years reported receiving an influenza and updated 2023–2024 COVID-19 vaccine, respectively; 17.0% of adults aged ≥60 years had received RSV vaccine. Coverage varied by demographic characteristics. Overall, approximately 27% and 41% of adults aged ≥18 years and 53% of adults aged ≥60 years reported that they definitely or probably will be vaccinated or were unsure whether they would be vaccinated against influenza, COVID-19, and RSV, respectively. Strong provider recommendations for and offers of vaccination could increase influenza, COVID-19, and RSV vaccination coverage. Immunization programs and vaccination partners are encouraged to use these data to understand vaccination patterns and attitudes toward vaccination in their jurisdictions to guide planning, implementation, strengthening, and evaluation of vaccination activities.

## Introduction

Influenza, SARS-CoV-2, and respiratory syncytial virus (RSV) typically circulate in the United States during the fall through early spring each year, causing epidemics of respiratory illness, although patterns of influenza and RSV transmission shifted during the COVID-19 pandemic (*1*–*3*). Certain groups, including older adults (those aged ≥65 years), persons with chronic conditions, and racial and ethnic minority populations, have experienced disproportionate influenza-, COVID-19–, and RSV-associated morbidity and mortality (*1*–*4*). Since 2010, the Advisory Committee on Immunization Practices (ACIP) has recommended routine annual influenza vaccination for all persons aged ≥6 months who do not have contraindications (*1*). On September 12, 2023, ACIP recommended updated 2023–2024 COVID-19 vaccination for all persons aged ≥6 months to help protect against currently circulating SARS-CoV-2 variants (*2*). In June 2023, ACIP recommended that adults aged ≥60 years may receive a single dose of RSV vaccine, using shared clinical decision-making, which is the first time a vaccine for prevention of RSV-associated respiratory disease has been recommended* (*3*). CDC monitors coverage with these vaccines and makes these data available during the respiratory season for use in planning vaccination activities.

## Methods

### Data Collection

The National Immunization Survey-Adult COVID Module (NIS-ACM) is a random-digit–dialed cellular telephone survey of adults aged ≥18 years in all 50 states, the District of Columbia, and selected local areas and U.S. territories. Data are weighted to represent the noninstitutionalized U.S. population.^†^ The survey includes questions about receipt of COVID-19, influenza, and RSV vaccines, vaccination intent, sociodemographic characteristics, and behavioral and social drivers of COVID-19 vaccination. Respondents are asked if they have received a COVID-19 or RSV vaccine or have received an influenza vaccine since July 1, 2023, and for affirmative responses, the month and year of vaccination.^§^ Those reporting receipt of any COVID-19 vaccine since September 14, 2023, are considered to be vaccinated with the updated 2023–2024 COVID-19 vaccine, because this was the only COVID-19 vaccine authorized in the United States after that date. 

### Data Analysis

Data collected during September 24–December 9, 2023, are included in this analysis.^¶^ Estimates of coverage (percentage of the population vaccinated) with influenza, COVID-19, and RSV vaccines were calculated for weekly data collection periods using a nondecreasing composite estimation procedure that uses data from completed interviews from the current week combined with data from all previous weeks (*5*). Estimates for vaccination intent are based on interviews conducted each respective week and are adjusted to the cumulative vaccination coverage estimate for that week. Influenza and COVID-19 vaccination coverage is estimated among adults aged ≥18 years, and RSV vaccination coverage estimates are restricted to respondents aged ≥60 years. Differences among estimates were determined using t-tests with p<0.05 considered statistically significant. This activity was reviewed by CDC, deemed not research, and was conducted consistent with applicable federal law and CDC policy.**

## Results

### Overall Vaccination Coverage and Intent

**FIGURE 1:**
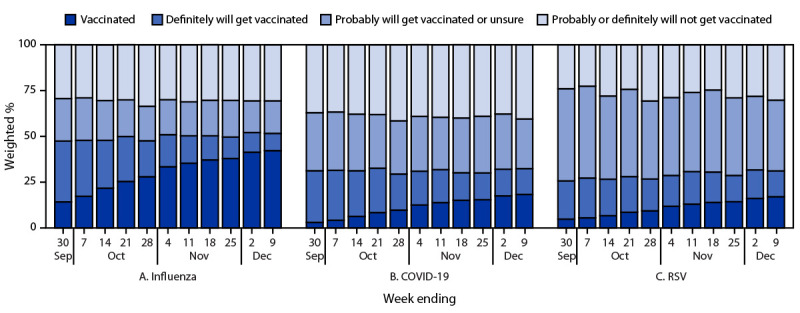
Weekly influenza (A), updated COVID-19 (B), and respiratory syncytial virus (C) vaccination status* and vaccination intent^†^ among adults^§^ — National Immunization Survey-Adult COVID Module, United States, September 24–December 9, 2023 **Abbreviation:** RSV = respiratory syncytial virus. * Estimates of vaccination coverage were calculated for December 3–9, 2023 using a nondecreasing composite estimation procedure that uses data from all completed interviews during September 24–December 9, 2023: influenza (168,899), COVID-19 (168,669), and RSV (62,816). ^†^ Estimates for vaccination intent are based on interviews conducted during December 3–9, 2023, and were adjusted to the cumulative vaccination coverage estimate for that week: influenza (14,562), COVID-19 (14,539), and RSV (5,258). Estimates for vaccination intent are not shown for groups with sample size <30. ^§^ Estimates for influenza and COVID-19 vaccination coverage and vaccination intent are among adults aged ≥18 years. Estimates for RSV vaccination coverage and intent are among adults aged ≥60 years.

As of December 9, 2023, estimated influenza and updated COVID-19 vaccination coverage among adults aged ≥18 years was 42.2% and 18.3%, respectively; estimated RSV vaccination coverage among all adults aged ≥60 years was 17.0% and among those with chronic health conditions^††^ was 21.4% (Figure 1) (Supplementary Table, https://stacks.cdc.gov/view/cdc/136452). From September 24 through December 9, the percentage of adults who reported being unvaccinated, but who definitely will get vaccinated, decreased over time as vaccination coverage increased, from 33.2% to 9.4% for influenza and from 28.2% to 14.1% for COVID-19 vaccines. The decrease was less for RSV vaccine (from 20.9% to 14.1%). Throughout the study period, the proportion of adults who were unvaccinated and reported they probably or definitely would not get vaccinated was lowest for RSV, whereas the proportion who were unvaccinated and reported they probably would get vaccinated or were unsure was highest for RSV.

### Vaccination Coverage and Intent by Demographic Characteristics and Jurisdiction

**FIGURE 2:**
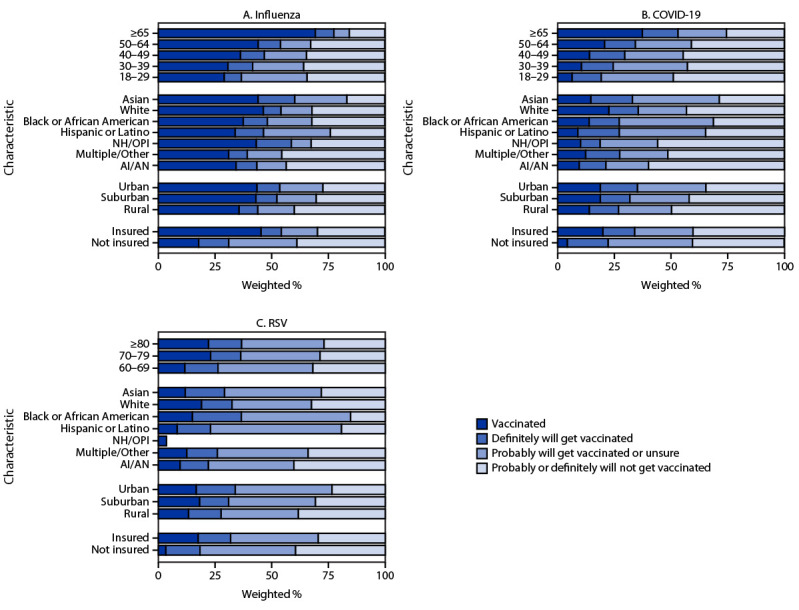
Influenza (A), updated COVID-19 (B), and respiratory syncytial virus (C) vaccination status* and vaccination intent^†^ among adults,^§^ by demographic characteristics^¶^ — National Immunization Survey-Adult COVID Module, United States, December 3–9, 2023 **Abbreviations:** AI/AN = American Indian or Alaska Native; NH/OPI = Native Hawaiian or other Pacific Islander; RSV = respiratory syncytial virus. * Estimates of vaccination coverage were calculated for December 3–9, 2023 using a nondecreasing composite estimation procedure that uses data from all completed interviews during September 24–December 9, 2023: influenza (168,899), COVID-19 (168,669), and RSV (62,816). ^†^ Estimates for vaccination intent are based on interviews conducted during December 3–9, 2023, and were adjusted to the cumulative vaccination coverage estimate for that week: influenza (14,562), COVID-19 (14,539), and RSV (5,258). Estimates for vaccination intent are not shown for groups with sample size <30. ^§^ Estimates for influenza and COVID-19 vaccination coverage and vaccination intent are among adults aged ≥18 years. Estimates for RSV vaccination coverage and intent are among adults aged ≥60 years. ^¶^ Persons of Hispanic or Latino (Hispanic) origin might be of any race but are categorized as Hispanic; all racial groups are non-Hispanic.

Coverage with all vaccines was lowest among uninsured persons. Coverage and intent to be vaccinated increased with age and were higher among adults living in urban and suburban areas compared with those living in rural areas (Figure 2). Influenza vaccination coverage was higher among non-Hispanic White (White) and non-Hispanic Asian (Asian) adults than among most other racial and ethnic groups. However, the percentage of persons reporting that they probably or definitely will not get an influenza vaccination was similar among White adults (32.2%) and Black or African American (Black) adults (32.2%) and was lower among Hispanic or Latino (Hispanic) adults (24.0%). Updated COVID-19 and RSV vaccination coverage was higher among White adults than among most other racial and ethnic groups. However, a higher percentage of White adults reported that they probably or definitely will not receive a COVID-19 vaccine (43.2%) than did Black (31.3%) and Hispanic (34.7%) adults. Similarly, a higher percentage of White adults reported that they probably or definitely will not receive an RSV vaccine (32.5%) than did Black (15.3%) and Hispanic (19.3%) adults. Coverage with all vaccines varied by jurisdiction, ranging from 15.6% to 54.8% for influenza vaccine, from 2.4% to 35.6% for updated COVID-19 vaccine, and from 1.9% to 32.4% for RSV vaccine (Table).

**TABLE:** Coverage with influenza, updated COVID-19, and respiratory syncytial virus vaccines among adults,* by jurisdiction — National Immunization Survey-Adult COVID Module, United States, September 24–December 9, 2023

Jurisdiction	Influenza		COVID-19		RSV	
	Cumulative unweighted no.	% Vaccinated (95% CI)^†^	Cumulative unweighted no.	% Vaccinated (95% CI)^†^	Cumulative unweighted no.	% Vaccinated (95% CI)^†^
Alabama	4,015	39.1 (35.4–42.8)	4,010	11.4 (9.3–13.4)	1,740	12.1 (8.4–15.7)
Alaska	2,079	39.7 (35.3–44.2)	2,075	16.0 (13.0–19.0)	734	21.3 (16.1–26.6)
Arizona	5,023	41.0 (37.5–44.5)	5,017	17.7 (15.4–20.1)	2,129	22.0 (17.4–26.6)
Arkansas	2,599	42.2 (37.0–47.3)	2,591	14.5 (11.1–17.8)	1,037	14.7 (9.7–19.8)
California	4,232	44.9 (40.6–49.2)	4,227	20.0 (16.8–23.1)	1,300	16.4 (10.5–22.3)
Colorado	3,686	49.1 (45.0–53.3)	3,680	25.7 (22.3–29.2)	1,308	32.4 (25.9–39.0)
Connecticut	1,235	47.0 (39.2–54.8)	1,233	23.6 (16.1–31.0)	212	24.1 (11.1–37.1)
Delaware	2,530	51.2 (43.9–58.6)	2,527	23.1 (17.7–28.6)	1,194	17.2 (12.6–21.8)
District of Columbia	4,307	52.9 (49.2–56.5)	4,304	35.6 (32.2–38.9)	1,291	20.7 (16.3–25.2)
Florida	2,045	36.0 (30.0–41.9)	2,043	10.9 (7.8–14.1)	733	20.7 (13.0–28.4)
Georgia	1,132	33.3 (25.3–41.3)	1,132	11.2 (5.1–17.3)	222	9.4 (5.2–13.7)
Hawaii	3,323	46.1 (42.0–50.1)	3,319	20.1 (17.2–22.9)	1,421	19.2 (14.9–23.5)
Idaho	1,474	34.8 (29.7–39.9)	1,472	16.5 (12.9–20.2)	502	21.0 (13.9–28.2)
Illinois	7,190	48.0 (44.9–51.2)	7,175	24.6 (22.0–27.2)	2,662	20.3 (16.7–23.8)
Indiana	2,678	39.5 (35.7–43.3)	2,671	16.8 (13.9–19.6)	1,003	14.5 (11.2–17.9)
Iowa	1,510	46.1 (38.3–53.9)	1,509	26.8 (18.7–34.9)	583	22.9 (7.3–38.5)
Kansas	2,875	44.7 (39.6–49.7)	2,871	20.0 (16.1–23.8)	960	22.4 (15.0–29.8)
Kentucky	2,290	39.6 (32.6–46.7)	2,287	14.8 (8.8–20.7)	804	22.9 (7.7–38.2)
Louisiana	3,719	36.8 (33.0–40.5)	3,713	10.1 (8.0–12.1)	1,542	13.8 (10.4–17.2)
Maine	4,291	49.2 (42.6–55.9)	4,287	28.8 (22.7–34.8)	1,949	18.0 (11.7–24.4)
Maryland	2,263	46.7 (41.2–52.2)	2,261	24.7 (17.7–31.7)	471	29.3 (17.2–41.3)
Massachusetts	4,696	50.7 (47.5–54.0)	4,689	28.4 (25.8–31.0)	1,764	23.9 (18.7–29.1)
Michigan	1,080	43.6 (35.4–51.7)	1,079	18.1 (12.2–23.9)	226	13.8 (5.2–22.4)
Minnesota	3,154	48.0 (44.6–51.4)	3,149	31.5 (28.4–34.5)	1,316	19.3 (15.2–23.4)
Mississippi	2,663	32.2 (27.9–36.5)	2,663	7.3 (4.9–9.8)	1,106	10.8 (6.0–15.6)
Missouri	1,211	43.3 (34.9–51.7)	1,213	21.1 (14.4–27.7)	315	14.1 (2.8–25.3)
Montana	3,301	39.4 (34.8–44.0)	3,292	20.9 (16.9–24.8)	1,510	17.1 (12.0–22.2)
Nebraska	1,990	47.9 (40.7–55.2)	1,986	18.7 (12.5–24.9)	715	20.7 (10.4–31.0)
Nevada	4,243	34.6 (31.7–37.4)	4,234	14.9 (12.9–16.9)	1,678	19.7 (15.9–23.5)
New Hampshire	4,620	51.0 (47.4–54.7)	4,619	27.6 (24.6–30.7)	2,276	17.6 (14.6–20.7)
New Jersey	3,780	45.7 (41.6–49.9)	3,770	19.1 (16.2–22.0)	1,350	14.6 (9.3–19.8)
New Mexico	4,041	44.5 (40.9–48.1)	4,034	19.8 (17.3–22.2)	1,596	23.3 (19.3–27.3)
New York	5,101	43.0 (39.7–46.2)	5,093	16.3 (14.3–18.4)	1,419	10.8 (8.2–13.5)
North Carolina	4,240	43.9 (40.3–47.4)	4,234	18.3 (15.6–21.0)	1,618	16.0 (12.4–19.6)
North Dakota	1,880	43.9 (39.5–48.2)	1,877	17.7 (14.6–20.8)	684	16.8 (10.4–23.2)
Ohio	1,148	44.1 (36.5–51.7)	1,148	17.5 (12.2–22.7)	206	19.9 (9.3–30.5)
Oklahoma	4,872	39.8 (36.7–42.9)	4,866	13.6 (11.7–15.5)	1,938	17.3 (13.9–20.8)
Oregon	3,080	40.8 (37.1–44.5)	3,078	25.0 (21.6–28.5)	1,200	20.3 (15.8–24.7)
Pennsylvania	8,446	43.4 (40.8–46.0)	8,432	19.8 (17.8–21.7)	3,217	14.4 (10.3–18.6)
Puerto Rico	4,742	28.3 (25.8–30.8)	4,735	5.3 (4.2–6.4)	1,911	4.1 (2.3–6.0)
Rhode Island	894	44.4 (36.6–52.2)	895	27.0 (19.1–35.0)	138	10.9 (2.0–19.7)
South Carolina	2,951	42.5 (36.6–48.3)	2,955	16.7 (11.9–21.4)	1,285	10.1 (7.3–12.9)
South Dakota	3,794	49.4 (45.5–53.3)	3,790	20.0 (17.4–22.6)	1,711	15.5 (12.2–18.9)
Tennessee	855	35.6 (27.7–43.4)	853	11.4 (5.8–17.0)	183	8.8 (2.4–15.3)
Texas	9,153	40.1 (34.9–45.4)	9,136	15.2 (11.0–19.4)	2,964	14.6 (10.2–19.0)
U.S. Virgin Islands	1,782	15.6 (12.5–18.6)	1,784	2.4 (1.5–3.3)	876	1.9 (0.9–2.9)
Utah	854	41.2 (33.7–48.7)	852	15.0 (9.7–20.3)	175	19.9 (4.4–35.4)
Vermont	831	54.8 (44.9–64.8)	831	32.0 (24.9–39.0)	164	21.8 (10.5–33.0)
Virginia	5,900	47.5 (45.0–50.1)	5,890	22.4 (20.5–24.3)	2,034	18.5 (15.5–21.5)
Washington	1,445	41.5 (35.0–48.0)	1,440	21.7 (15.9–27.6)	278	20.2 (12.0–28.4)
West Virginia	1,886	44.8 (35.9–53.7)	1,888	16.4 (9.6–23.2)	771	7.7 (5.5–10.0)
Wisconsin	2,690	46.4 (41.8–51.0)	2,684	23.7 (20.1–27.3)	1,054	14.3 (10.3–18.2)
Wyoming	3,080	35.6 (32.3–38.8)	3,076	14.9 (12.7–17.0)	1,341	16.6 (12.8–20.5)
**Range across jurisdictions**	**—**	**15.6–54.8**	**—**	**2.4–35.6**	**—**	**1.9–32.4**

**Abbreviation:** RSV = respiratory syncytial virus.

* Estimates presented for influenza and COVID-19 vaccination are among adults aged ≥18 years. Estimates for RSV vaccination are among adults aged ≥60 years. Estimates of vaccination coverage were calculated for December 3–9, 2023 using a nondecreasing composite estimation procedure, which uses data collected from all completed interviews during September 24–December 9, 2023: influenza (168,899), COVID-19 (168,669), and RSV (62,816).

^†^ Weighted percentage.

## Discussion

As of December 9, 2023, self-reported coverage with influenza, updated COVID-19, and RSV vaccines among U.S. adults was low, particularly for updated COVID-19 and RSV vaccines. RSV vaccination coverage was low even among persons with chronic conditions who are at highest risk for severe RSV disease and might benefit from vaccination. As of mid-November, influenza vaccination coverage was approximately 2.5 percentage points lower than it was at the same time during the 2022–23 influenza season (*6*). Approximately 41% of all adults and 53% of adults aged ≥60 years were unvaccinated but reported that they definitely or probably plan to receive or are unsure about receiving updated COVID-19 and RSV vaccines, respectively, suggesting they are open to vaccination. A health care provider recommendation for and offer of vaccination are strongly associated with vaccination (*7*). A previous report found that unvaccinated adults who were open to receiving a bivalent COVID-19 vaccine had not yet done so mainly because of concerns about side effects, being too busy, or just had not gotten around to getting vaccinated (*8*). Making vaccination available in provider offices, pharmacies, workplaces, and other convenient locations at convenient times, along with a strong provider recommendation for vaccination, could increase vaccination coverage, particularly for RSV, which is recommended on the basis of shared clinical decision-making between a patient and provider (*3*).

Despite disparities in vaccination coverage by race and ethnicity, when responses indicating the person is open to vaccination are included, the potential vaccination coverage that could be achieved for Hispanic, Black, and Asian adults is similar to or higher than that for White adults. Programmatic measures that helped reduce disparities in coverage with the primary series of COVID-19 vaccine, such as making vaccines available free of charge, use of trusted messengers, and bringing vaccines into communities through nontraditional settings (e.g., local libraries and local businesses such as barber shops and restaurants)^§§^ (*4*,*9*), might increase equitable access to vaccination and decrease disparities for these currently recommended vaccines.

CDC is partnering with community-based organizations, health care providers, and other trusted messengers to build vaccine confidence and awareness, including through the Partnering for Vaccine Equity program.^¶¶^ CDC is also working to expand COVID-19 vaccine access to all through the Bridge Access Program, which provides COVID-19 vaccines for adults without health insurance and adults whose insurance does not cover all COVID-19 vaccine costs. Public health safety net and pharmacy locations offering influenza and COVID-19 vaccines, including COVID-19 vaccines through the Bridge Access Program, are available at https://www.vaccines.gov. Communication campaigns,*** such as the “Wild to Mild” and “Get My Flu Shot” influenza vaccine campaign and the “Everything” broad respiratory virus communication initiative, include various materials and resources to promote vaccination, including to persons who are disproportionately affected by disease. Finally, CDC has developed health care provider toolkits to empower providers with knowledge to confidently recommend vaccination.^†††^

CDC makes vaccination coverage estimates rapidly available during the respiratory virus season.^§§§^^,^^¶¶¶^ In addition to data from the NIS-ACM, vaccination data are available from multiple sources and include coverage among children, pregnant persons, Medicare beneficiaries, and national projected vaccination in pharmacies and medical offices. Jurisdiction-level estimates of COVID-19 vaccination coverage and intent stratified by demographic factors, behavioral and social drivers of vaccination, and barriers to vaccination are available.**** CDC’s COVID-19 Vaccination Geographic Information System Mapping Tool, designed with feedback from several local health departments, provides web maps where jurisdiction-level data including demographic characteristics and social determinants of health can be displayed along with vaccine confidence and vaccination coverage.^††††^ End-of-season influenza vaccination coverage estimates for children and adults since the 2010–11 influenza season, nationally and by state, are available on FluVaxView.^§§§§^

### Limitations

The findings in this report are subject to at least three limitations. First, response rates for NIS-ACM were relatively low (<25%). Data were weighted to mitigate possible bias resulting from incomplete sample frame (i.e., exclusion of households with no phone service or only landline telephones) or nonresponse, but some selection bias might persist. Second, all responses were self-reported; vaccination receipt, and month and year of receipt of most recent dose might be subject to recall or social desirability bias. Nonresponse and social desirability bias could result in overestimation of coverage. Third, the survey sampled noninstitutionalized U.S. adults; therefore, adults who were incarcerated or who live in long-term care facilities^¶¶¶¶^ might not be represented in the sample.

### Implications for Public Health Practice

Although influenza, updated COVID-19, and RSV vaccination has slowed for the 2023–24 respiratory season, vaccination is recommended to continue while viruses are circulating (*1**–**3*), and many unvaccinated persons continue to report intent to be vaccinated. Health care provider recommendations for and offers of vaccination are important to increasing vaccination coverage (*7*). Immunization programs and vaccination partners are encouraged to use CDC developed dashboards and tools, as well as other data sources available to them, such as immunization information systems, to identify undervaccinated populations and better understand vaccination patterns, attitudes and behaviors, and systemic barriers to vaccination in their jurisdiction to help tailor vaccination activities to improve coverage and health equity.

## Footnotes

* On September 22, 2023, ACIP and CDC recommended maternal Pfizer RSVpreF vaccination for pregnant persons as a one-time dose at 32–36 weeks’ gestation using seasonal administration (i.e., September–January in most of the continental United States) for prevention of RSV-associated lower respiratory tract infection among infants aged <6 months. Either maternal RSVpreF vaccination during pregnancy at 32–36 weeks’ gestation or nirsevimab (a human recombinant monoclonal antibody) immunization for infants aged <8 months who are born during or are entering their first RSV season is recommended, but administration of both products is not needed for most infants. (http://dx.doi.org/10.15585/mmwr.mm7241e1). Information about RSV vaccination during pregnancy and nirsevimab receipt among infants is not included in this report.

† Data were weighted to represent the noninstitutionalized U.S. population aged ≥18 years using population control totals for age group, sex, metropolitan statistical area status, education, Hispanic origin, and race. https://www.cdc.gov/vaccines/imz-managers/nis/about.html

§ Respondents reporting receipt of an influenza vaccine since July 1, 2023, are asked the month and year of first vaccination since July 1, 2023. Respondents reporting receipt of ≥1 dose of any COVID-19 vaccine are asked the month and year of most recent COVID-19 vaccination; if the reported month of vaccination is September 2023, they are further asked if they received a COVID-19 vaccine since September 14, 2023. Those reporting receiving any COVID-19 vaccine since September 14, 2023, are considered vaccinated with the updated 2023–2024 COVID-19 vaccine. Respondents reporting receipt of RSV vaccine are asked the month and year that they received the RSV vaccine.

¶ Respondents were excluded from the analysis if they did not answer the question or questions about receipt of vaccine or intent to be vaccinated, and for RSV, if they did not report age. Number of excluded respondents was 365 for influenza vaccination coverage assessment, 595 for COVID-19 vaccination coverage assessment, and 3,890 for RSV vaccination coverage assessment. Sample sizes for data collected through December 9, 2023, and included in this analysis were 168,899 for influenza vaccination assessment, 168,669 for COVID-19 vaccination assessment, and 62,816 for RSV vaccination assessment.

** 45 C.F.R. part 46.102(l)(2), 21 C.F.R. part 56; 42 U.S.C. Sect. 241(d); 5 U.S.C. Sect. 552a; 44 U.S.C. Sect. 3501 et seq.

†† Includes persons who reported any of the following chronic conditions: chronic lung disease, diabetes, heart conditions, kidney disease, liver disease, or weakened immune system.

§§
https://www.cdc.gov/vaccines/health-equity/field-stories.html

¶¶
https://www.cdc.gov/vaccines/health-equity/

***
https://www.cdc.gov/flu/resource-center/toolkit/index.htm

††† https://www.cdc.gov/respiratory-viruses/tools-resources/health-care-providers.html; https://www.cdc.gov/vaccines/covid-19/; https://www.cdc.gov/vaccines/vpd/rsv/hcp/older-adults.html; https://www.cdc.gov/flu/professionals/vaccination/prepare-practice-tools.htm

§§§ https://www.cdc.gov/respiratory-viruses/data-research/dashboard/snapshot.html (Accessed December 15, 2023).

¶¶¶
https://www.cdc.gov/vaccines/imz-managers/coverage/respvaxview/index.html

**** Data on updated 2023–2024 COVID-19 vaccine will be added January 24, 2024. https://www.cdc.gov/vaccines/imz-managers/coverage/covidvaxview/interactive.html

††††
https://www.cdc.gov/vaccines/imz-managers/coverage/covidvaxview/covid19-vaccination-mapping-tool.html

§§§§
https://www.cdc.gov/flu/fluvaxview/coverage-by-season.htm

¶¶¶¶ https://www.cdc.gov/mmwr/volumes/72/wr/mm7251a3.htm?s_cid=mm7251a3_w
